# Social media best practices for the spine care professional

**DOI:** 10.1016/j.xnsj.2025.100748

**Published:** 2025-06-16

**Authors:** Zachary A. Cupler, Andrew Trontis, Brandon Lucke-Wold, Samuel M. Schut, Khoi D. Than, James E. Eubanks, Robert J. Butler, Reem Elwy, David Gendelberg

**Affiliations:** a Physical Medicine and Rehabilitative Services, Butler VA Health Care System, Butler, PA, United States; b Institute for Clinical Research Education, University of Pittsburgh, Pittsburgh, PA, United States; c Progressive Spine & Orthopaedics, Edgewood, NJ, United States; d Department of Neurosurgery, University of Florida, Gainesville, FL, United States; e Department of Wellness and Preventive Medicine, Cleveland Clinic, Cleveland, OH, United States; f Cleveland Clinic Lerner College of Medicine of Case Western Reserve University School of Medicine, Cleveland, OH, United States; g Department of Neurosurgery, Duke University, Durham, NC, United States; h Department of Orthopaedics and Physical Medicine and Rehabilitation, Division of Physical Medicine and Rehabilitation, Medical University of South Carolina, Charleston, SC, United States; i Department of Physical Medicine and Rehabilitation, University of Pittsburgh Medical Center, Pittsburgh, PA, United States; j Integrated Primary Care, VA Palo Alto Health Care System, Palo Alto, CA, United States; k Department of Neurosurgery, Cairo University, Cairo, Egypt; l Department of Orthopaedic Surgery, Orthopaedic Trauma Institute, University of California San Francisco, San Francisco, CA, United States

**Keywords:** Social media, Spine care, Best practices, Guidelines, Ethics, Professionalism, Chiropractic, Physical therapy, Neurosurgery, Orthopedics

## Abstract

**Background:**

Physicians, allied health clinicians, scientists, and industry partners’ use of social media has grown exponentially, permeating across all levels of healthcare delivery and practice. Spine care professionals can interact with both professional and patient audiences across social media platforms.

**Methods:**

This article aims to narratively outline social media best practices for the spine care professional.

**Results:**

Thirty-two social media best practice statements are presented to help guide spine care professionals. The best practice statements are thematically organized: (1) Compliance and Confidentiality, (2) Professionalism, (3) Security and Access, and (4) Spine Care.

**Conclusions:**

Social media represents a broadcasting platform for communication between clinicians and patients alike, presenting with it numerous advantages and challenges. As spine care professionals we must learn how to engage in its utilization in both educational and professional-social environments.

## Introduction

Multidirectional communication is common on the internet today due to the development and popularity of social media applications (i.e., X [formerly Twitter], Facebook, Instagram, Tik Tok, LinkedIn, YouTube). Social media enables user-generated content, content sharing, and community development [1]. Among health care professionals, social media use has increased exponentially in recent years [[2], [3], [4], [5], [6], [7], [8]]. This global, interactive communication tool has created the ability for spine care professionals (i.e., chiropractors [[9], [10], [11], [12]], neurological and orthopedic spine surgeons [2,13,14], physiatrists [15], physical therapists [16,17], and research scientists) and trainees [[18], [19], [20], [21]] to connect with and distribute information to both lay and professional audiences on unprecedented scale. Recently, relatively few policies are available to provide guidance for social media use among spine care professionals [22].

Challenges exist in the ethical and professional uptake of social media use by spine care professionals because of this lack of guidance. Ethical conundrums and concern for privacy errors or missteps with social media use are perhaps reasons for slower uptake by health care professionals when compared to the public [23,24]. As spine care professionals, posting original content or sharing other’s content is expected to be accurate and concordant with best practices reported in the literature. At times, dubious and inconsistent misinformation is shared on social media [[9], [10], [11],[25], [26], [27], [28]]. For example, acute low back pain TikTok videos created by clinicians generally did not provide self-care information consistent with guidelines [25]. A majority of YouTube videos for spine condition management are poor to moderate in quality and are discordant with clinical guidelines [[29], [30], [31], [32]]. Some spine care professionals may have concerns about violating Health Insurance Portability and Accountability Act (HIPAA) if informed consent was not properly acquired to share patient information including personally identifiable information or images on social media, which could lead to serious consequences [33].

The objective of this best practices document is to provide an up-to-date contemporary view of spine care professional conduct and decorum for social media platforms. The goal of these best practices is to assist spine care professionals in ethically maintaining confidentiality, privacy, public trust, and collegiality on social media platforms while avoiding the pitfalls of unethical behavior.

## Methods

We performed an iterative consensus process for social media best practices statements for the spine care professional after a review of the literature through May of 2024.

This project is the product of the North American Spine Society’s (NASS) Early Career Advisory Council, an interdisciplinary working group of early career spine care professionals that included chiropractors, neurosurgical and orthopedic spine surgeons, physiatrists, physical therapists, and clinical and basic science researchers.

We included searches of PubMed and Google related to social media, code of conduct, policy, and best practices. These best practice recommendations were then synthesized from the literature search and the interdisciplinary work group’s experience. The best practice statements were revised through an iterative process and approved by authors. Key domains were identified and used to structure the spine care professional social media use best practice statements.

## Results

One organizational code of conduct [34], one organization’s recommendations [35]. Nine peer-reviewed articles [2,16,[36], [37], [38], [39], [40], [41], [42]] (Table 1) were identified as relevant. The American Medical Association: Code of Medical Ethics [34] described 7 principles. In summary, familiarity with patient privacy and confidentiality standards transcends all environments including online. Physicians are to maintain comparable physician-patient relationship boundaries to in office care and abide by confidentiality, privacy and informed consent standards. Should content posted by colleagues breach professional norms, appropriate action is necessary such as reporting to licensing boards. Finally, physician and physician trainee online behavior has repercussions to public trust for both the individual and the medical profession reputations.

**Table 1:** Summary table of included peer reviewed manuscripts.

Reference (Last Name, Year)	Specialty or audience	Study design	Objective	Key take aways
Chidharla, 2022 [38]	Oncology	Literature review	Discuss how various forms of social media can be used for continuous professional development and academic promotion and characterize the risks associated with social media use in oncology.	Guidance on the professional use of social media - Be intentional—think before you post. Aim to add something constructive. - Be transparent—proactively disclose relationships outside of your current position, not reactively. - Be clear—speak plainly and clearly. Your audience is global, not medical. - Be smart—do not post anything you may regret. Do not post when you are angry, exhausted, or inebriated - Be yourself—be authentic. Interact as yourself. Remember, how much the public learns about you is up to you.

Farnan, 2013 [36]	American College of Physicians	Position paper	Provide guidance for practitioners, trainees, and medical students in navigating the digital world, including the use of social networking, blogging, online forums, media sharing sites, cell phone photography, electronic searching, texting, and e-mailing.	*Position 1*: Use of online media can bring significant educational benefits to patients and physicians, but may also pose ethical challenges. Maintaining trust in the profession and in patient–physician relationships requires that physicians consistently apply ethical principles for preserving the relationship, confidentiality, privacy, and respect for persons to online settings and communications. *Position 2*: The boundaries between professional and social spheres can blur online. Physicians should keep the 2 spheres separate and comport themselves professionally in both. *Position 3*: E-mail or other electronic communications should only be used by physicians in an established patient physician relationship and with patient consent. Documentation about patient care communications should be included in the patient’s medical record. *Position 4*: Physicians should consider periodically “self-auditing” to assess the accuracy of information available about them on physician-ranking Web sites and other sources online. *Position 5*: The reach of the Internet and online communications is far and often permanent. Physicians, trainees, and medical students should be aware that online postings may have future implications for their professional lives.

Hennessy, 2019 [37]	Australian Medical Association and New Zealand Medical Association, British Medical Association, General Medical Council, Canadian Federation of Medical Students, American College of Physicians, Federation of State Medical Boards, American Medical Association, Canadian Medical Association and faculty members	Thematic analysis	Summarize the guidelines provided by medical governing bodies from major international English-speaking countries on social media use and provide practical advice on how social media can be used.	Five common themes - Accountability and responsibility to maintain public’s trust in profession. - Warning that expected privacy and anonymity is not guaranteed. - Confidentiality of patient information. - Defamation of patients, colleagues, and products. - Encouragement and reasons to use social media.

Gagnon, 2015 [16]	Physical therapy	Commentary: Perspective	Review of the emergence of social media in society and health care, explores policy implications of organizational adoption of health care social media, and proposes individual opportunities and guidelines for social media use by the physical therapy professional.	Potential, practical approaches and guidelines for social media use by individual health care professionals should be more than a set of “do’s and dont’s” and must articulate more than avoidance of negative behaviors. Rather, guidelines should provide recommendations for a thoughtful, effective social media presence that adheres to platform-specific terms of service, professional standards, and organizational policies. Social Media Guidelines for Health Care Providers - Have clear objectives. - Never share patient-specific information. - Educate yourself on the policies of your employer and adhere to them. - Be yourself and declare conflicts of interest. - Practice digital professionalism. - Control information sharing. - Take an agnostic approach to social media. - Monitor your online identity.

Guraya, 2021 [39]	Healthcare trainees and professionals	Systematic review	To review the available body of knowledge that can help identify key concepts and threats to professional identity in the era of digital professionalism.	Forty-four articles met criteria for inclusion. There are unprofessional behaviors on social media among health care professionals. There is a high prevalence of breach of patient confidentiality due to an absence of existing social media policies. Four main themes were generated from the review. - Usage of social media by health professionals and students. - The impact of social media on medical professionalism. - Blurring of professional values, behaviors, and identity in the digital era. - Teaching and assessing professionalism in the digital era.


Kind, 2015 [41]	Healthcare professionals	Commentary	None	Guidelines applicable to professional conduct in “offline” in person settings can also provide a useful model for how we should conduct ourselves online. The “starting point” should always be our existing norms of communication, confidentiality, and all the relevant tenets of professionalism, applied to new settings. We should retain the principles underlying norms of professionalism and apply them to new contexts. Physicians should maintain their integrity; compassion; respect for others; responsiveness to patient needs that supersedes self-interest; respect for privacy and autonomy; accountability to patients, society, and the profession; sensitivity to diverse populations; and commitment to ethical principles regarding care, confidentiality, informed consent, and business practices irrespective of “when” and “where.”

Samtani, 2023 [2]	Spine surgery	Cross-sectional	To document the social media presence of a broad cohort of spine surgeons and to discuss the benefits and risks of a social media presence.	For 325 Spine Surgeons from 76 institutions across the US, 64.6% of spine surgeons had at least 1 professional social media profile: LinkedIn (57.2%), Facebook (17.8%), Practice Website (15.7%), Twitter (13.8%), Instagram (7.1%), or YouTube (6.5%) Benefits of social media use to a spine practice - Marketing to patients and practice building - Patient education - Professional education

Vukušić Rukavina, 2021 [42]	Healthcare professionals	Scoping review	Characterize e-professionalism of health care professionals and provide insights to guide future research in this area.	A total of 88 studies were included for evaluation. All studies were exploratory in nature, and the findings were descriptive.Benefits of social media on e-professionalism of health care professionals: - Professional networking and collaboration. - Professional education and training. - Patient education and health promotion. Dangers of social media on e-professionalism of health care professionals: - Loosening accountability. - Compromising confidentiality. - Blurred professional boundaries. - Depiction of unprofessional behavior. - Legal issues and disciplinary consequences.

Vukušić Rukavina, 2022 [40]	Medical and dental trainees and faculty	Mixed methods	Characterize web-based professionalism on publicly available Facebook profiles of medical or dental students and faculty.	Unprofessional or potentially unprofessional content on medical or dental students and faculty public Facebook profile can be classified. The level of web-based professionalism on Facebook profiles of medical or dental students and faculty available for public viewing shows a high level of understanding of e-professionalism, with unprofessional content being very low.

The Federation of State Medical Boards Ethics and Professionalism Committee provided sixteen recommendations for physicians [35]. Maintenance of in person decorum and patient-physician boundaries was emphasized. The committee also highlighted the permanency of the post and lack of control one has with their content once it is posted to social media. Although, not social media specific, the committee found it important is their recommendations to describe decorum for management of artificial assistant devices since as Amazon’s Alexa device in clinical areas—recommending to turn off the setting so the device will not record patient health information.

**Fig. 1:**
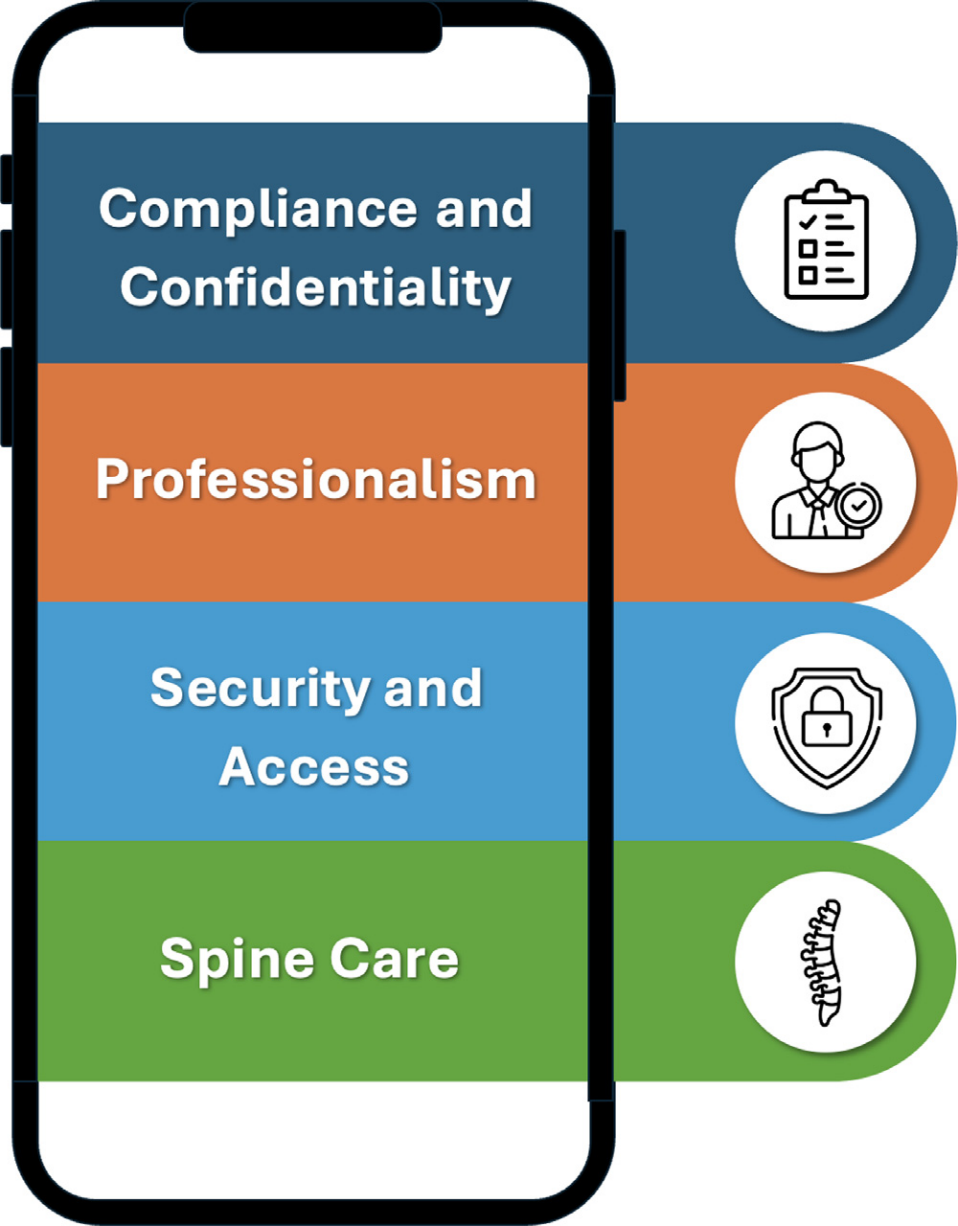
Four key themes identified for social media best practices for spine care professionals. (Created by Samuel Schut, DC).

These included papers and codes were used to support the best practices. Four key themes were identified for social media best practices for spine care professionals: (1) Compliance and confidentiality, (2) Professionalism, (3) Security and Access, and 4) Spine Care Considerations (Fig. 1). Social media guidance statements for the spine care professional were synthesized from the included papers and documents and the work group opinion. Thirty-two best practices statement were derived for spine care professionals.

### Compliance and Confidentiality

Spine care professionals should take careful consideration to present themselves in a transparent and accurate manner. Consistent with in-person services at a medical facility, credentials should be readily visible and great care should be taken to maintain patient confidentiality. There were eleven best practice statements specific to compliance and confidentiality are further organized to “account information” and “compliance.”

For account information, (1) clearly and accurately represent level of training, qualifications, and professional credentials (e.g., alluding that one is board certified when they are not). (2) It is important to plainly state that information posted on the account represents only the account owner’s views and do not represent those of their employer or institution. This may include a formal disclaimer such as “*Content or opinions represent (spine care professional’s name) views, and not those of (employer)*.”

Regarding compliance, (3) social media accounts should conform to employer and/or institutional social media policy. (4) HIPAA and Food and Drug Administration (FDA) compliance is applicable to social media posts. (5) Check photos for protected health information (PHI) prior to “posting” or “sharing” pictures and images to social media. (6) Do not use the camera or share screen function to record videos or join live events (i.e., conference calls) where patient or PHI is visible (i.e., surgical suite, ambulatory center, outpatient clinic).

(7) Be familiar with your state professional licensing board or other governing bodies’ policies regarding social media conduct and behavior. (8) Do not post about specific patients, even in general terms, when informed consent has not been explicitly received to share information on social media. (9) If a patient has given their expressed, written permission for you to use their information (i.e., HIPAA authorization), include a clear statement indicating you have received this permission.

(10) If comments or posts from other sources on the social media account may include or lead to community or legal violations, content should be moderated or removed (i.e., deleting comments, locking a post from comments, ensuring PHI is not shared). (11) Further, if comments are allowed on your social media account, consider developing a transparent policy outlining instances in which you as the account owner may delete or otherwise moderate the content posted (i.e., comments) on your profile or account: a) This may include, but are not limited to, posts that include inappropriate language and promotion of specific products or companies, b) Ensure consistent application of these policies across all platforms, and c) Adherence to national, state, and institutional policies regarding comment discussions.

### Professionalism

Consistent with face-to-face professional boundaries, spine care professionals should not provide medical advice via social media but rather focus on education and awareness. Social media should be assumed to be part of a permanent record and content shared on the spine care professional’s account should reflect consistency with the literature and/or best practice. There were 6 best practice statements specific to professionalism.

Regarding professionalism, (1) maintain professional boundaries with both patients, the public, and colleagues (i.e., clinician and nonclinician) when communicating through social media. (2) Limit engagement with patients on social media platforms and redirect to private secure platforms when indicated. If a patient or public member does reach out regarding medical advice, redirect them to an official communication channel (i.e., office phone number, medical office portal system).

(3) Be cognizant of “re-posting,” “sharing,” and “liking” of content from specific companies or products which may be construed as promotion or endorsement of that company or product. If there is potential for confusion, the spine specialist should clarify that the “posting” or “sharing” does not necessarily represent endorsement. (4) Be mindful of social media demeanor and consider anything posted as an enduring statement. To this end, prepare all social media content as if it is a professional speaking engagement. Assume anything posted will reflect on you, your colleagues, spine care at large, and may have implications for the entirety of your career. (5) Pause before “posting” content to social media to allow time to reflect upon the appropriateness of your prepared content. (6) Slander and defamation of other spine care or healthcare professionals should be avoided.

### Security and access

Data protection and social media account security is paramount for spine care professionals. Strong password development, use of 2-factor authentication, and established institution based information technology practices to maintain passkeys should be applied to social media accounts. There were 4 best practice statements specific to professionalism.

For security and access, (1) maintain separate personal and professional social media accounts. (2) Ensure strong password controls, especially if other office staff will have access to professional social media profile accounts. (3) Develop and maintain an employee and staff social media policy for professional account management that includes detailed steps for appropriate conduct and repercussions when standard practices are breached. (4) If staff with access leaves employment (i.e., moves to another department/position, resigns), promptly remove access to professional accounts.

### Spine care

There are unique considerations specific to spine care that encompass the variety of health disciplines ranging from physicians, allied health clinicians, complementary integrative health clinicians, and scientific researchers. There were eleven best practice statements specific and distinctive to spine care.

Regarding specific spine care social media guidance, (1) recognize the interprofessional nature of spine care and avoid disparaging language and comments directed at the variety of spine care management strategies. (2) Give mutual respect among the various expertise and experiences of spine care professionals. (3) Employ disability-inclusive language when discussing persons with spine conditions and refer to those conditions by their acceptable medical terminology.

(4) Consider the appropriateness and professionalism of demonstrating spine care treatments (i.e., surgery, interventional procedures, spinal manipulation, and therapeutic exercise) in photos and videos. (5) When capturing images or videos of evaluation, procedural, or operative techniques of patients, avoid capturing identifying information as well as tattoos and other identifiable markings when possible. (6) Be mindful of social media content creation used for ‘click bait’ (i.e., young attractive models, tight fitting clothing, video angles) that would downplay the professionalism of spine care professionals.

(7) Patient testimonies for spine care treatments should clearly state or identify if the patient did or did not receive compensation (i.e., monetary compensation, exchange of services) for their testimony. (8) A statement should be applied to a post (i.e., text, video, image) when there is any potential conflict of interest including direct or indirect compensation or services exchanged for product placement, sponsored posts, and product endorsements on a spine care professional’s social media account.

(9) Consider the alignment and credibility of original and ‘shared’ reposted content with clinical practice guidelines, best practices, and available peer-reviewed evidence to avoid misinforming the public. This includes regular evaluation of enduring content and citing relevant literature when available. (10) Consider the appropriateness of sensationalizing treatments (i.e., hyperbolic language, postvideo production modifications, amplification of sound) with demonstration videos. (11) Verify the credibility of the material (text, image, or video content), check the date of the information to ensure current relevance, and cite the source which may include hyperlinking to the original content. This helps ensure copyright compliance prior to “sharing,” “liking,” or “posting” content from another source.

## Discussion

We describe, to our knowledge, the first interdisciplinary spine care professional social media use best practices that included the perspectives of chiropractors, neurosurgical and orthopedic spine surgeons, physiatrists, physical therapists, and clinical and basic science researchers. Social media use for the spine care professional should adhere to aspects of medical ethics: (1) beneficence—the need to act in the best interest of the patient (*Salus aegroti suprema lex*); (2) nonmaleficence—“first, do no harm” (*primum non nocere*); and (3) respect for persons—the patient has the right to be treated with dignity and honesty [43]. Each and every spine care professional should take necessary steps to optimize the presented social media use best practices. For example, a patient is likely to recognize images or videos of themselves even with removal of PHI. Considering several best practice statements, sharing images or video of a specific patient’s case should only be posted with the informed consent of the patient.

Our recommended best practices align with existing guiding documents, but in contrast with the American Medical Association and the Federation of State Medical Boards, we address specific concerns that arise across the range of professionals involved in spine care, research, and training [34,35]. Though uncertainties remain, it is a professional obligation for spine care professionals to use social media platforms in a safe, ethical, and accurate manner with the best interest of the patient placed at the center [12]. We find our best practices recommendations congruent and aligned with a recent expert opinion social media behavior guideline from the American Society of Pain and Neuroscience NEURON Project which generated similar recommendations to consider legal and ethical considerations and priority to disseminate accurate and digestible online content [44]. Appropriate social media best practices should be distributed to spine care professionals and trainees during undergraduate, graduate, and postgraduate spine care training.

Spine care professionals have numerous opportunities to improve public knowledge of spine-related disorders and patient care outcomes. Original and shared content vetted by health care professionals ensures credible and accurate spine care information accessible to the public, filling a consumer void for high-quality online information on spine care, health, and rehabilitation [16,45]. For example, some information on operative spine procedures posted by spine surgeons might overemphasize and extrapolate postoperative imaging outcomes without providing a comprehensive clinical scenario that might inadvertently mislead future patient expectations [14]. In another study assessing TikTok videos for guideline recommended acute nonspecific low back pain, nearly 50% of content creators were influencers or fitness professionals, indicating there is a need for high quality and accurate spine care professional-generated content [25].

Condition specific information (i.e., scoliosis [46] or spinal stenosis [47]) that is accurate and reflective of the current literature can be responsibly shared and subsequently accessed by the public who may not have the specialized skill sets necessary to evaluate primary literature. For example, a recent intentionally designed social media campaign, “*Let’s Talk Turkey About Low Back Pain,*” was organized by the pain collaborative to advance equitable value-based solutions to leverage existing professional networks to raise public awareness by amplifying messaging of nonspecific low back pain [48]. Quality of care at hospitals, ambulatory centers, and outpatient clinics may be associated with engagement with social media but not necessarily in a positive direction [[49], [50], [51], [52]]. For example, 1 study found that the number of Facebook “Likes” was negatively associated with 30-day mortality and positively associated with recommendations for care [53].

Employing disability-inclusive language in social media affords spine care professionals an opportunity to elevate discussions about and with those with spine-related disabilities. Spine care professionals need to consider the language used when referring to patients and those with spine-related conditions in social media posts. In 2019, the United Nations Office of Geneva released guidelines as part of their United Nations Disability Inclusion Strategy for the implementation of disability-inclusive language [54]. These guidelines are “aimed at removing barriers and engaging persons with disabilities in all spheres of work and life in order to achieve sustainable and transformative progress on disability inclusion.” Notably, the recommendations intend to inform oral and written communications, including social media.

Research, professional connections, and spine care training programs have immense reach through social media platforms. Social media interaction among professional peers at medical conferences can engage, connect colleagues, and create community during virtual meetings [55,56]. Investigators can turn to social media platforms to conduct well-represented international surveys of the spine care field [57,58]. Research dissemination through social media has a global reach and serves as a real-time or asynchronous connection between spine care professionals that spans disciplines, institutions, and countries. Understanding the patient experience as shared on social media is a rich tapestry of content that continues to evolve [59]. Finally, spine care training programs can directly interface with potential candidates for advanced training and residencies [60,61]. An assessment of orthopedic residency programs found nearly one third of included programs maintained program-affiliated Facebook, Instagram, and X (formerly Twitter) accounts. [20] With social media, prospective applicants can get a feel for an institutional atmosphere and foster virtual connections with current trainees and faculty.

## Limitations

This document is not intended for any one specific social media platform and is intended to be generalizable across all platforms. Artificial intelligence (AI) was considered beyond the scope of this social media use best practices for the spine care professional as the applicability is rapidly changing. In future spine care professional social media best practices, the application of AI in social media should be considered.

We caution that these best practices are not intended to expand or restrict a health care clinician’s scope of practice or to supersede applicable ethical standards or provisions of law. Spine care professionals should weigh several considerations when creating and maintaining a social media presence and ultimately should uphold the same professional conduct expected in face-to-face interactions.

The nature of social media is a double-edged sword, meaning potential benefits and pitfalls, remains an emerging technology as spine care professionals learn how to engage in its utilization in both training and professional-social environments. Spine care professionals’ uptake and utilization of social media is not well understood and is an area for future investigation. Social media is evolving rapidly, and these best practices are not intended as a fixed document but are guiding principles. It is anticipated that there will be rapid transformation and novel applications discovered that will have the potential to improve health education, health outcomes, productivity, and professional dialogue with spine care professionals, the public, and patients. We anticipate future modifications and revisions to these best practices as new technology emerges.

## Conclusion

Social media best practices for the spine care professional are challenging and nuanced. Engagement with social media to communicate with the public presents a quandary, presenting an opportunity to present up-to-date health information while also potentially exposing oneself to ethical violations. These best practices for social media for the spine care professional will promote safe and ethical use of social media.

## Disclaimers

This is not an official position of the North American Spine Society.

The views expressed in this article are those of the authors and do not reflect the official policy or position of the Department of Veterans Affairs, or the United States Government.

## Prior presentations

Material from this manuscript was presented at the XXXV Congresso Brasileiro de Neurocirurgia, Belo Horizonte, Brazil, September 6, 2024.

## Declarations of competing interest

One or more of the authors declare financial or professional relationships on ICMJE-NASSJ disclosure forms.
